# Introduction: selected extended articles from the 2nd International Workshop on Semantics-Powered Data Analytics (SEPDA 2017)

**DOI:** 10.1186/s12911-018-0624-8

**Published:** 2018-07-23

**Authors:** Zhe He, Cui Tao, Jiang Bian, Rui Zhang, Jingshan Huang

**Affiliations:** 1 School of Information, Florida State University, 142 Collegiate Loop, Tallahassee, 32306 FL USA; 2 School of Biomedical Informatics, University of Texas Health Science Center at Houston, Houston, TX USA; 3 Department of Health Outcomes and Biomedical Informatics, University of Florida, Gainesville, FL USA; 4 Institute for Health Informatics and College of Pharmacy, University of Minnesota, Minneapolis, MN USA; 5 School of Computing, University of South Alabama, Mobile, AL USA

**Keywords:** Health data analytics, Ontology, Data mining, Natural language processing

## Abstract

In this editorial, we first summarize the 2nd International Workshop on Semantics-Powered Data Analytics (SEPDA 2017) held on November 13, 2017 in Kansas City, Missouri, U.S.A., and then briefly introduce 13 research articles included in this supplement issue, covering topics such as Semantic Integration, Deep Learning, Knowledge Base Construction, and Natural Language Processing.

## Background

In the past decade, a variety of health data has been generated and collected from hospitals, national surveys, online social media platforms, mobile devices, and fitness wearables. In 2009, the Health Information Technology for Economic and Clinical Health (HITECH) Act, enacted as part of the American Recovery and Reinvestment Act of 2009, was signed into law. The purpose of the HITECH Act is to promote the adoption and meaningful use of health information technology such as electronic health records (EHR) for eligible healthcare professionals and hospitals. HITECH’s multi-stage meaningful use criteria ensure that providers are using certified EHR technology in ways that can be measured both qualitatively and quantitatively [1]. As of December 2017, about 90% of the office-based physicians in the United States are using EHRs [2]. Moreover, in the past few years, various clinical data research networks such as the National Patient-Centered Clinical Research Network (PCORnet) [3], Observational Health Data Sciences and Informatics (OHDSI) [4] have been established with common data models, allowing researchers to conduct large-scale aggregate analysis of clinical data across disparate systems to advance medical knowledge and improve population health. However, a major limitation of these clinical research data networks built from the clinical data is that they mostly contain data for patients who have visited hospitals or clinics, but not people who are healthy or do not visit healthcare organizations. In 2016, President Obama launched the Precision Medicine Initiative (now called “All of Us Research Program”) with a $215 million investment to understand how a person’s genetics, environment, and lifestyle can help determine the best approach to prevent or treat disease. This program will enroll about one million patients from across the United States to donate their clinical and genomic data to enable previously infeasible research for a wide range of common and rare diseases, meanwhile ensuring the statistical power for detecting associations between environmental and/or biological exposures and a wide variety of health outcomes. In addition, according to a recent report conducted by Researchscape International [5], over half of Americans (51%) reported using a wearable fitness tracker at least once a day. As such, fitness wearables are an increasingly common method to collect data from a broader population outside of the hospital setting.

The technological advancements in artificial intelligence, cloud computing, and computing power pose unprecedented opportunities for mining previously undiscovered knowledge from the dramatically increasing amount of healthcare data. However, due to the disparities in different health data sources, it is still challenging to fully exploit their value and effectively mine useful information from both structured and unstructured data to solve real-world health problems, such as disease phenotyping and risk factor identification. We believe that it is time that researchers in various domains work together and develop novel methodologies and algorithms to tackle this challenging problem in health data analytics.

## SEPDA workshop

Various healthcare information systems such as EHRs have integrated well-curated biomedical controlled vocabularies, e.g., the International Classification of Diseases (ICD) and RxNORM, as their vocabulary foundation [6]. With rich medical concepts linked by hierarchical and associative relationships, these vocabularies and ontologies can also be utilized in health data analytics tasks such as natural language processing, data integration, and decision support [7]. Opportunities exist for leveraging semantic methods to enhance these data science efforts. In December 2016, we held the First International Workshop on Semantics-Powered Data Analytics (SEPDA 2017) in Shenzhen, China. In October 2017, we published a special issue in the Journal of Healthcare Engineering on “Semantics-Powered Healthcare Engineering and Data Analytics” [8]. In November 2017, we successfully held SEPDA 2017 in Kansas City, Missouri, in conjunction with IEEE 2017 International Conference on Bioinformatics and Biomedicine (BIBM 2017). From the SEPDA 2017 workshop, we selected and invited 13 high-quality submissions to extend their workshop papers for this journal supplement.

This supplement aims to showcase the state-of-the-art research and development efforts that effectively use biomedical ontologies and/or semantics methods to address important problems in health and biomedicine. The selected papers underwent a rigorous review and revision process. We are glad to see that the selected papers present novel usage of semantics-based techniques and ontologies in natural language processing, deep learning, pattern mining, semantic integration, and knowledge base construction, while addressing key problems in healthcare. Meanwhile, we have also included foundational research papers that are focused on developing and curating biomedical ontologies. These studies can be categorized into the following three topics: (1) natural language processing, (2) data mining and deep learning, and (3) ontologies, semantic integration, and knowledge bases. We introduce them as follows.

## Natural language processing

Information extraction in EHRs is an applied field within NLP. Such applications usually focus on the information absent from structured data but stored in unstructured clinical reports, retrieval of which requires the aid of natural language processing techniques. For example, in the paper “*Discovering and identifying New York Heart Association (NYHA) classification from electronic health records*,” Zhang et al. identified potential sources of NYHA in a local EHR system and reported that the richest source of NYHA class is clinical notes [9]. They then demonstrated that a random forest classifier with n-gram features can extract NYHA classes from clinical notes. Similarly, dietary supplement use information is usually stored in the clinical reports in EHRs, thus extracting such information is significant for supporting supplement safety surveillance and subsequent research. In the paper “*Using natural language processing methods to classify use status of dietary supplements in clinical notes*.” Fan et al. developed and compared NLP methods to extract and classify use status of dietary supplement in clinical reports. They reported that a machine learning-based model outperformed a rule-based method in a relatively large corpus [10].

Relationship extraction is another active field in clinical NLP. Identifying relationship between a time expression and an event mention from clinical texts is essential to understand a patient’s conditions; however, most work showed low performance for identifying both explicit and implicit relationships. This makes it challenging to identify temporal relationships for practical use. In the paper “*Identifying direct temporal relations between time and events from clinical notes*,” Lee et al. focus on the identification of direct temporal relationships, which contain important information needed for clinical applications [11]. They developed a system achieving better performance than the state-of-the-art system developed on a comprehensive set of temporal relationships. Identifying interactions between proteins in the biomedical literature is another application of relationship extraction. In the paper “*Automatic extraction of protein-protein interactions using grammatical relationship graph*,” Yu et al. developed an approach based on NLP and graph theoretic algorithm to extract the protein-protein interactions [12]. The method shows a higher precision compared with other methods.

## Data mining and deep learning

With health information technologies broadly implemented, large collections of longitudinal data can be leveraged for secondary use. Big health data has been used in many clinical research applications such as discovering new disease association and personalized treatment with data mining techniques. Due to the richness and heterogeneity of biomedical and clinical data, mining useful information is challenging. In this respect, customized data mining techniques and deep learning have shown the potential to accelerate knowledge discovery and information extraction from aggregated multidimensional data. Deep learning not only avoids the labor intensive feature engineering process but also shows competitive performance.

In the paper “*Chemical-induced disease extraction via recurrent piecewise convolutional neural networks*,” Li et al. developed a novel document-level recurrent piecewise convolutional neural network for chemical-induced disease extraction [13]. The authors used the Chemical-induced Disease Relation dataset in BioCreative V and demonstrated performance that is competitive with other state-of-the-art systems.

Neural word embeddings have recently been used as inputs in many text classification tasks with promising performance. However, the semantic relationships in the neural word embeddings have not been sufficiently investigated. In the paper “*Evaluating semantic relations in neural word embeddings with biomedical and general domain knowledge bases*”, the authors used WordNet and the Unified Medical Language System as the gold standard to assess the semantic relationships in the word embeddings generated by Word2vec and its variations such as GloVe [14].

Image processing has been another popular application of deep learning but computer-aided image processing and diagnosis systems have not yet been widely adopted. In the paper “*Towards improving diagnosis of skin diseases by combining deep neural network and human expertise*”, Zhang et al. used deep learning algorithms to help diagnose four common cutaneous diseases based on dermoscopic images [15]. Besides promising accuracy of diagnosis, their semantic summarization of diagnosis scenarios can also help further improve the algorithm to facilitate computer-aided decision support.

Social media has been recently recognized as an important source for early detection of mental health issues. In the paper “*Extract psychiatric stressors for suicides from social media using deep learning*”, Du et al. compared a convolutional neural network based algorithm with traditional machine learning algorithms to extract psychiatric stressors from Twitter data [16]. According to their findings, deep learning based approaches showed superior performance over traditional machine learning algorithms.

In the paper, “*Query-constraint-based mining of association rules for exploratory analysis of clinical datasets in the National Sleep Research Resource*,” Abeysinghe and Cui introduced a query-constraint-based association rule mining approach for the exploratory analysis of five clinical datasets in the National Sleep Research Resource [17]. By merging similar variables and removing general rules, their algorithm can generate concise and interesting rules, some of which were validated by evidence from biomedical literature.

## Ontology, semantic integration, and knowledge bases

Ontologies are widely used in biological and biomedical research, where the past two decades have witnessed an explosion of ontology developments, from the development of the Gene Ontology (GO) in 1998 to national coordination efforts such as the Open Biomedical Ontologies (OBO) Foundry and the National Center for Biomedical Ontology. The characteristics of ontologies make them an ideal knowledge representation tool for both humans and computers. Ontologies standardize the vocabularies used within a domain, thus, a big portion of ontology research is to develop domain-specific ontologies. For example, in the paper titled “*Visualized Emotion Ontology: a model for representing visual cues of emotions*” by Lin et al., the authors created the Visualized Emotion Ontology (VEO) to provide semantic definitions of 25 emotions based on the revised Ortony, Clore, & Collins’ model of emotions [18]. What was novel about their study is that they not only created a machine-understandable artifact but also linked it with human-friendly visualizations.

As a knowledge representation tool, ontologically structured data of a specific domain are often being referred to as a knowledge base (KB). How to automatically curate these KBs from free-text data is also an active research area. In the paper from Lossio-Ventura et al., “*OC-2-KB: Integrating crowdsourcing into an obesity and cancer knowledge base curation system*,” they integrated a crowdsourcing process into their existing natural language processing and machine learning-based KB curation system, which has significantly improved the system’s performance and subsequently the quality of the curated knowledge [19]. One of the primary uses of ontologies is to enable data integration across multiple databases, where the use of standard identifiers for classes and relations in ontologies is a key component. In Zhang et al.’s work, “*An ontology-guided semantic data integration framework to support integrative data analysis of cancer survival*,” they created a semantic data integration framework to address the syntactic, schematic, and semantic heterogeneities when linking disparate data sources to produce pooled datasets for downstream data analysis needs [20]. In the work, “*A semantics-oriented computational approach to investigate microRNA regulation on glucocorticoid resistance in pediatric acute lymphoblastic leukemia*”, Chen et al. demonstrated the utility of a semantic search tool based on integrated biomedical and biological ontologies to help better investigate microRNA regulation mechanisms in Glucocorticoids-resistant pediatric acute lymphoblastic leukemia patients [21].

## Discussion and conclusion

The ever-growing amount of digital health data pose significant challenges for effective data analytics, semantic integration, and decision support. The multidisciplinary nature of this supplement is reflected by the fact that challenging problems in semantics-powered data analytics are being tackled by teams of researchers in different disciplines with diverse expertise. Novel methods have been devised to tranform disparate health data into actionable knowledge to improve healthcare. We envision these work will have significant impact on health and biomedical informatics. It is our hope that more researchers will be empowered by these work to contribute and advance the field in the near future.
